# Using change detection to objectively evaluate whether novel over-the-counter drug labels can increase attention to critical health information among older adults

**DOI:** 10.1186/s41235-021-00307-z

**Published:** 2021-05-26

**Authors:** Alyssa L. Harben, Deborah A. Kashy, Shiva Esfahanian, Lanqing Liu, Laura Bix, Mark W. Becker

**Affiliations:** 1 Michigan State University School of Packaging, 448 Wilson Road, East Lansing, MI 48824 USA; 2 Department of Psychology, Michigan State University, 316 Physic Rd., East Lansing, MI 48824 USA

## Abstract

Over-the-counter (OTC) drugs have many benefits but also carry risks, such as adverse drug reactions, which are more prevalent in older adults. Because these products do not require the oversight of a physician or pharmacist, labeling plays a key role in communicating information required for their safe and effective use. Research suggests that current labels are not terribly effective at communicating potential risk. One reason for their lack of effectiveness is that few consumers attend to critical information (active ingredients and warnings) when making purchases. In two experiments, we used a change detection task to objectively evaluate how novel label designs that employ highlighting and a warning label placed on the package’s front impact attention to critical information among older participants (65 and older). The change detection task is a unique form of visual search which allowed us to assess the attentional priority of critical information among participants who were not explicitly instructed to search for this critical information. This unique aspect of the task is important given research suggesting that consumers rarely have the explicit goal of seeking out warnings and active ingredients when making OTC selections. Our results provide empirical support that both highlighting critical information and positioning it on the package’s front increase its attentional prioritization relative to current, commercial practice. Given that attending to the critical information is prerequisite to utilizing that information, strategies that elicit attention in this way are likely to reduce medication errors.

## Significance

Over-the-counter (OTC) medicines provide many benefits, including increased access, independence, flexibility, and affordability. As a result, they are widely used. Despite their advantages, OTCs carry significant risks for Adverse Drug Reactions (ADRs) (O’Connor et al., 2012). It has been estimated that 106,000 US deaths per year are attributable to an ADR (Lazarou et al., 1998), and a Canadian study estimates the cost of emergency and hospital care associated with ADRs at $35,700,000 (USD) annually (Wu et al., 2012), more than half of which were thought to be preventable. Among those who experienced ADRs that resulted from self-administration, between a third (Asseray et al., 2013) and half (Schmiedl et al., 2014) were attributable to OTCs, as opposed to prescription medications.

Because OTC medication selection and use are not guaranteed to be informed by a prescribing physician or dispensing pharmacist, the creation of labels that facilitate processing of information critical for their safe and effective use is paramount. In the United States, fairly comprehensive regulations (Labeling Requirements for over-the-counter Drugs, 2020) mandate the content and formatting of OTC labels. However, research suggests that few consumers pay attention to current warning labels when making drug selections, with many never turning beyond the package’s front when deciding on a product’s appropriateness for their use (Liu, 2016; McNeil Consumer Healthcare, 2015). Here, we test novel OTC labels designed to increase attention to critical warnings and active ingredient information. A change detection method was used to investigate whether these labels increase attention to this information among older adults. Results provide potential insights for designers and regulators, indicating that highlighting critical information and the use of a front warning label can increase the attentional prioritization of information that is critical to the safe and effective use of OTC medications in an at-risk population.

## Introduction

Visual search tasks have a long history in cognitive psychology. While most early experiments utilized these tasks to investigate basic mechanisms of attention (Schneider & Shiffrin, 1977; Treisman & Gelade, 1980; Wolfe, 1994), in recent years there has been an increased emphasis on leveraging our knowledge of visual search to real world applications. Many of the early attempts to do so focused on real world activities such as radiology (Wolfe et al., 2016, 2017) and baggage screening (Biggs & Mitroff, 2014; Mitroff et al., 2015), tasks that, at least on the surface, closely mimic typical lab-based visual search tasks. Given that attention to information is a prerequisite for processing and using that information (Mack, 2003; Rensink et al., 1997; Simons & Chabris, 1999), there are a myriad of scenarios which less closely mirror traditional lab-based studies where visual search tasks can be used to investigate how well critical information attracts attention. Application of visual search tasks in these spaces provide a powerful method to objectively evaluate the relative performance of various design strategies intended to increase attention to critical information. For instance, our group has leveraged visual search tasks to evaluate how different strategies for designing labels influence attention to the most critical information presented on the label. We have done this for prescription drug labels (DeHenau et al., 2016; Lee et al., 2019), food labels (Becker et al., 2015), and medical device labels (Seo, 2014).

Here, we use a modified form of visual search, the change detection method, to investigate whether novel labeling for OTC medications can increase attention to critical label information, namely the active ingredients and warnings specific to that drug that should be attended to ensure safe usage (specifically, warnings about drug-drug and drug-diagnosis interactions). There are a number of reasons why we believe this change detection method is well-suited for our investigation.

First, while change detection with small arrays of stimuli has been used to evaluate visual working memory capacity (Luck & Vogel, 1997), change detection with more complicated scenes allows one to assess the attentional prioritization of elements of complex displays. For instance, one of the early reports suggesting that large changes in complex scenes were surprisingly difficult to detect also reported that changes to information that was semantically central to a scene were found readily (Rensink et al., 1997). This difference in detectability of changes based on their centrality to the scene, suggests that the time to detect a change could be used to evaluate when attention first reaches an object (Simons & Rensink, 2005), thereby providing a proxy for the attentional prioritization of specific elements of a complex display. Indeed, researchers have used time to detect a change as a metric of when attention first reaches an object. For instance, research has found that heavier social users of alcohol found changes to alcohol related objects more rapidly than light social users, suggesting that alcohol-related stimuli had higher attentional prioritization among those with higher usage (Jones et al., 2003). Others have used the time to detect changes to investigate how image properties influence the allocation of attention. For instance, changes to scene-inconsistent objects were detected more readily than changes to scene-consistent objects, suggesting that the inconsistencies drew attention (Stirk & Underwood, 2007). In sum, the change detection method allows one to objectively evaluate the attentional prioritization of elements within a complex visual stimulus.

Second, unlike traditional visual search tasks, the change detection method does not require participants to search for a specific search target. Instead, participants are simply asked to find a change anywhere that it occurs in the display. This unique aspect of the change detection task allows us to evaluate how our novel designs influence attentional prioritization of critical health information among observers who are not explicitly told to search for this critical information. This aspect of the change detection method is important because research suggests that few consumers intentionally seek-out and attend to this critical health information when evaluating medications (Liu, 2016; McNeil Consumer Healthcare, 2015). That is, typical consumers do not have the explicit goal of searching for critical health information; as such, evaluating the attentional prioritization of that information in the absence of such a goal is important to health, policy, and those designing these products. The change detection method allows us to evaluate attentional priority in the absence of an explicit goal to seek out this critical information, mimicking (to some degree) the typical consumer who is not intentionally looking for this information.

In short, this method provides a means of tracking the attentional prioritization of the active ingredient and warning information on OTC packages, while avoiding the need to inform people that warnings and active ingredients are of importance to our research. By systematically varying the format of the label and investigating how design changes impact attentional prioritization, we can identify labeling techniques that are effective at drawing attention to critical health information among participants, who like typical consumers, are not volitionally seeking out this information. Herein we manipulate OTC labels with two treatments: highlighting of critical information and the addition of a front warning label that contains critical information.

Highlighting was selected as a treatment because recent changes to the labeling regulations require highlighting or bolding of the active ingredient acetaminophen and the drug class NSAID (Specific Labeling Requirements for Specific Drug Products, 2020). The fact that regulators have, for the first time, included highlighting in the regulations suggest that this approach may be one that regulators would back. In addition, the adoption of those highlighting regulations was made without published research supporting the effectiveness of this strategy for OTC labeling. Thus, evaluating the effectiveness of the technique is timely and important.

The front warning label strategy was selected as a treatment due to the design strategy’s effectiveness in nutritional labeling (Becker et al., 2015; Bix et al., 2015; Borgmeier & Westenhoefer, 2009; Hersey et al., 2013; Jones & Richardson, 2007; Kelly et al., 2009; Roberto et al., 2012). We note that the nutrition facts label is similar to the drug facts label in both format and placement on a package. Thus, the effectiveness of a front-of-pack nutritional label makes it likely that such an approach may generalize to OTC drug labels. However, the front-of-pack approach has not been tested for OTC labelling.

## Background

**Fig. 1:**
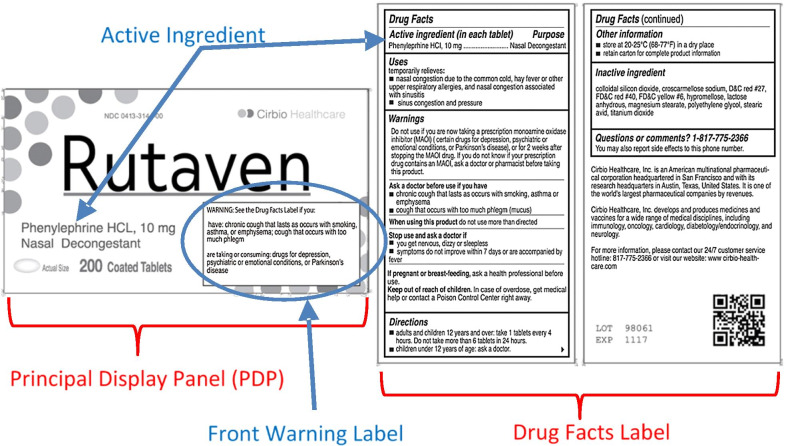
An example of one of the mock brands. For illustrative purposes, the principle display panel (PDP) and Drug Facts Label are labeled in red and the front warning label and active ingredients are labeled in blue. The actual stimulus did not show these colored labels, arrows, and circle

Over-the-counter (OTC) drugs have many benefits for consumers, but there are risks associated with their use, including adverse drug reactions (ADRs) (O’Connor et al., 2012). ADRs are defined as “any noxious, unintended and undesired effect of a drug, excluding therapeutic failures, intentional and accidental poisoning and drug abuse (O’Connor et al., 2012).” Because OTC medications don’t require the oversight of a learned intermediary (i.e. the prescribing physician or a dispensing pharmacist), the creation of labels that facilitate processing of information that is critical for their safe and effective use is paramount. As such, the US Food and Drug Administration (FDA) mandates both the content and formatting of the “Drug Facts Label", a comprehensive information panel present on most OTCs sold in US markets. The Drug Facts Label generally appears to the right of the Principal Display Panel, or PDP, defined as the panel that is customarily displayed at retail (see Fig. 1), or more commonly, the front panel of the package.

Objectively characterizing design strategies likely to catalyze consumers to attend critical information present on OTC labels is relevant to public health. Even so, both researchers and regulators guiding the development of OTC label formats tend to focus their efforts on late-stage processing (comprehension). Historically, comprehension studies have utilized surveys and questionnaires (Murty & Sansgiry, 2007; Wawruch et al., 2013); guided interviews (King et al., 2011; Martin-Hammond et al., 2015; Tong et al., 2015, 2017); and focus groups (King et al., 2011; Tong et al., 2016) in which participants are asked to evaluate which type of warning text or display would be more effective.

A weakness of relying on self-reports is that there is limited evidence supporting their validity in the OTC literature. That said, there is a body of literature that suggests that self-reported measures related to visual search can be problematic, particularly when people introspect about visual information they are likely to notice (Levin et al., 2000) or when the techniques are used as the basis for decision making (Morales et al., 2017; Nisbett & Wilson, 1977). Recently, researchers have begun to address this short-coming by using objective measures to evaluate OTC labels (see Tong et al., 2014 for a thorough review). However, even these approaches tend to focus efforts on comprehension (late-stage processing), with most studies based on tasks which explicitly require participants to attend to the critical information. This results in a gap in knowledge concerning whether the different methods of labeling critical information influence *attention* to the critical components of interest, or how design strategies garner (or fail to garner) attention absent an explicit goal which requires them to seek it.

Here, we empirically investigate how different label designs influence attention to critical information (active ingredients and/or warnings) using a change detection methodology. Specifically, we hypothesized that moving critical warnings to the front of the package and highlighting critical information, would be effective approaches for OTCs (see Fig. 1). The strategy of moving critical information to the front of the package has been found to be effective for food labels; when nutrition information related to disease (i.e. sodium, fat and sugar) are added to the front of the package they increase attention to these key elements (Becker et al., 2015; Bix et al., 2015). In fact, in food packaging a front of pack label has been shown to garner attention, speed cross-product comparisons (Borgmeier & Westenhoefer, 2009; Hersey et al., 2013; Jones & Richardson, 2007; Kelly et al., 2009; Roberto et al., 2012), and facilitate more healthful choices (Levy et al., 2012; Thorndike et al., 2012, 2014). The attentional benefits of front of package nutritional labels reported using change detection tasks (Becker, et al., 2015) have been replicated using eye tracking measures (Bix et al., 2015; Van Herpen & Van Trijp, 2011), including eye-tracking in a lab-based shopping market (Graham et al., 2015), providing some evidence that data from change detection tasks may generalize to more realistic purchase scenarios. In the current work we leveraged that nutrition research to design and objectively test whether moving critical warnings to the front of the package increases attention to critical information among participants that are not explicitly tasked with preferentially attending to this type of information.

We also evaluate whether highlighting critical information increases the attentional prioritization of this information. We chose to test highlighting because it has recently been embraced by regulators as a means of attracting attention to critical information, but its use is currently very limited. Acetaminophen was recently mandated to be *either* highlighted or bolded in its appearance in the PDP and the “Statement of Identity” section of the Drug Facts Label (it also *may* be highlighted or bolded in the Active Ingredients and Use Sections) (Specific Labeling Requirements for Specific Drug Products, 2020).^1^ The new requirements were likely based on recommendations from the “Acetaminophen Hepatoxicity Working Group,” which did not site empirical support for the approach (The Acetaminophen Hepatotoxicity Working Group, 2008). Our review of the literature uncovered limited work specific to the use of highlighting with OTC products. King et al. (2011) performed structured interviews and focus groups to investigate whether “patient-centered icons and messaging” could be used to enable consumers to identify the active ingredient acetaminophen. Although investigating highlighting was not their goal, they concluded that most participants wanted the word acetaminophen to be highlighted (King et al., 2011).

The fact that regulators have endorsed highlighting is promising. However, to date there is little empirical evidence supporting its effectiveness on OTC labels. In addition, we note that most of the empirical work on highlighting has been interested in understanding text comprehension in academic settings, and has found mixed results (see Dunlosky et al., 2013 for a review). However, that work seems to suggest that the advantage of highlighting is diminished when too much information is highlighted. Given the number of important warnings on OTCs is unclear whether highlighting would be effective or would be diluted by the shear amount of highlighting necessary.

In our experiments, we targeted older adults as participants because they are at increased risk from the ill-effects associated with ADRs. There are a variety of reasons for this increased risk, including: an increased propensity for poly pharmacy; (Davies & O’Mahony, 2015; Guthrie et al., 2015; Lavan & Gallagher, 2016); changes in pharmacodynamics and pharmacokinetics (Bourgeois et al., 2010; Lavan & Gallagher, 2016; Nair et al., 2016); as well as a tendency for lower health literacy (Davis et al., 2006; Kobayashi et al., 2016) and lower risk perception (McNeil Consumer Healthcare, 2015; Taylor, 1994; Wawruch et al., 2013) compared with younger adults. Specific to OTCs, older population are documented to use these medications at a higher and increasing rate (Qato et al., 2016), but have also been suggested as being less likely (than their younger counterparts) to report use of critical information that appears within the Drug Facts Label (McNeil Consumer Healthcare, 2015). Our study focuses on two types of critical information, active ingredients and warnings about possible drug-drug or drug-diagnosis interactions. These types of information were reported, in a national survey of pharmacists that we conducted (in preparation), as critical for the prevention of ADRs in older adults.

We fill an important gap in knowledge by empirically evaluating how the presence of a front warning label and highlighting affect the allocation of attention of older adults to information critical to the safe and effective use of OTCs when they perform a change detection task that does not require them to explicitly seek out critical health information. Although this method has been utilized with older adults to examine changes in visual attention while driving (Costello et al., 2010; Hoffman et al., 2005; McCarley et al., 2004; Pringle et al., 2001; Veiel et al., 2006), to our knowledge we are among the first to use it with older adults to assess the efficacy of labeling strategies with this population.

## Methods

### Participants

While no existing past research explicitly manipulates the variables in the present study within the context of OTC labels, our own work that used change detection to investigate the effects of front-of-pack nutrition labels (Becker et al., 2015) found that a sample size of 47 was more than adequate to find significant RT and accuracy effects of a front-of-pack and a color manipulation. We used that sample size as a benchmark and were conservative, anticipating a 20% attrition rate for this older sample, and thus recruited 60 participants for each experiment.

Participants were recruited from multiple locations in the state of Michigan, including: the greater Lansing area, Wayne County (Detroit), and Kent County (Grand Rapids). Recruitment was supported by the SONA system, campus-based email list serves, MSU Extension, and Wayne County’s Area Agency on Aging programs targeting seniors. Eligibility criteria included: age (65 +); the requirement of OTC use within the previous 12 months: being legally sighted; a history of purchasing and self-managing medication; a willingness and ability to travel for testing; and the capacity to render informed consent. Further, participants were screened for a history of epilepsy or seizures.

All methods were conducted in accordance with approvals granted by the MSU IRB under the number × 17-922eD (Experiment 1) and MSU Psychology and Social Science Internal Review Board STUDY00000832 (Experiment 2).

### Stimuli and design

**Fig. 2:**
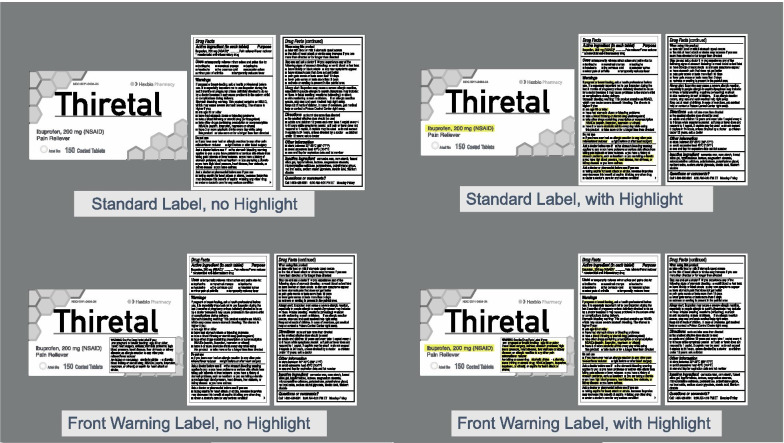
Examples of the four label treatments

We performed two experiments to investigate this issue. While there were minor differences between the two methods, the core of the experiments was the same. Both used a flicker change detection task to investigate how two design factors, highlighting and the use of a front warning label (see Fig. 2), influenced attention to critical information (specifically, active ingredients and the warnings previously mentioned). The flicker change detection task was a modification of the method used in Rensink et al. (1997). In both experiments, this involved cycling the following displays: a flattened image of a product label (240 ms), a blank gray image (80 ms), the original product label with some aspect of the label deleted (240 ms), and a blank gray image (80 ms). Participants were given 18 s to search for the change before the trial timed out and participants were prompted to move on to the next trial.

For the experiments, we made labels of mock brands of OTC medications, each mock brand had a single, unique active ingredient. All information on the packaging, including the Drug Facts Label, was based on commercially available products with a single active ingredient and complied with requirements dictated by the Code of Federal Regulations CFR Title 21 Subchapter 1 Part 201 Subpart C (less the highlighting requirement specific to Acetaminophen for non-highlighted treatments). Experiment 1 was programmed using E-prime Version 2, and Experiment 2 was programed used E-prime Version 3. Both were displayed at a resolution of 1920 × 1080 on 14″ screens.

We begin by walking through the detailed methods of Experiment 1 and then will discuss the modification that were made in Experiment 2. In Experiment 1, there were a total three mock brands, each treated a different ailment and had a different active ingredient. Each mock brand appeared 56 times for a total of 168 change trials. A given trial was classified, at the highest level, as either a critical change (*n* = 60) or a non-critical change (*n* = 108).

Non-critical changes occurred to graphics, or non-health critical information (e.g., the number of tablets, the manufacturer’s name). These non-critical trials were included to help ensure that the participants were not preferentially attending to warnings and active ingredient information. Again, we were interested in how our designs could impact attention to this critical information among those who were not seeking it out. If the changes consistently involved this critical information, participants would have likely notice this contingency and may have set a volitional goal of preferentially attending to this critical information, thereby defeating one of the purposes of using change detection; to evaluate prioritization among those who were not explicitly looking for warning and active ingredient information.

Non-critical trials provided two additional benefits. One was to avoid a bias toward critical information based on changes being concentrated at particular locations of the label. People are sensitive to spatial contingencies and will pay more attention to locations that are likely to contain targets (Geng & Behrmann, 2005; Miller, 1988) To avoid contingencies creating history effects (Awh et al., 2012) that might bias attention toward the locations of critical information, the non-critical trials were carefully counter-balanced to ensure that there were an equal number of changes in each of the locations of change (see Fig. 1—PDP and drug facts label), and that these were distributed throughout each. Finally, subjects’ average performance on non-critical trials were used as a co-variate in the analysis of critical trials, thereby allowing us to account for some of the between subject variability in overall performance.

**Fig. 3:**
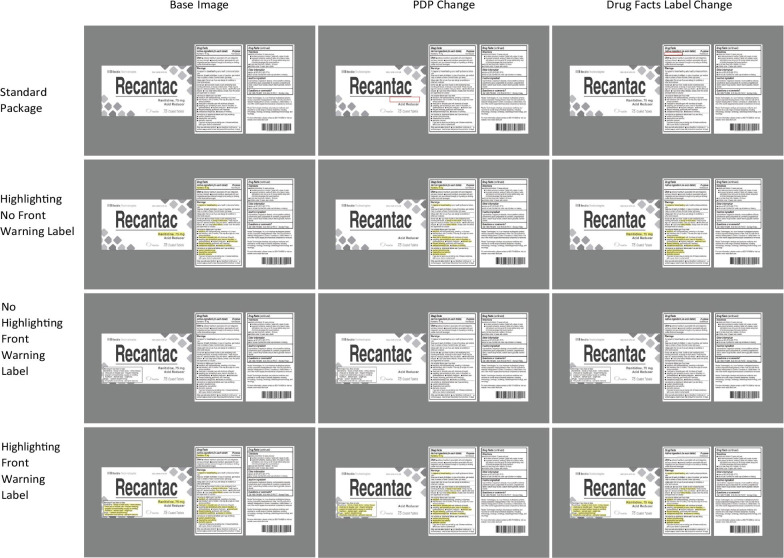
Examples of the eight active ingredient changes. The left column depicts the complete label for the 4 label treatments. The middle depicts the altered images with a change to the PDP. The right depicts the altered images with a change to the active ingredient in the Drug Facts Label. The red squares in the top row are to illustrate the change location and did not appear

Critical changes occurred to either the Active Ingredient (24 trials) or to a warning (36 trials). Within active ingredient changes the 24 trials were comprised of the full factorial combination of 3 mock brands × 2 locations of change (PDP or the Drug Facts Label) × 2 label conditions (a standard label or one with a front warning label) × 2 highlighting conditions (not highlighted or highlighted). For Experiment 1, in each of these trials the change involved the active ingredient repeatedly appearing and disappearing (see Fig. 3). In highlighted conditions, the active ingredient was highlighted on both the PDP and in the Drug Facts Label (the active ingredient typically appears in both locations), and key phrases within the warnings that indicated a drug/drug or drug/diagnosis warning were highlighted. When there was a front of pack warning, the same warning text was highlighted in both locations (PDP and Drug Facts Label).

Ideally, across the eight trials within a brand, the size of the changing information would be held constant. For a given location (e.g., PDP or Drug Facts Label) this was accomplished; the same exact active ingredient appeared and disappeared once in each condition for each brand. However, when comparing across location of change (PDP vs Drug Facts Label), size was not identical. Based on current regulations and practice, the active ingredient is typically presented in a larger font on the PDP than in the Drug Facts Label. To be consistent with regulations and practice, we maintained this difference. As a result, direct comparisons of changes to active ingredients on the Drug Facts Label to changes in active ingredients on the PDP, could be due to the differences in size. However, we note that prior research suggests that the importance, or centrality, of the change rather than its size influences the ease of change (Rensink et al., 1997; Stirk & Underwood, 2007). Further, we note that this confound *does not* occur for the warning changes (see below).

**Fig. 4:**
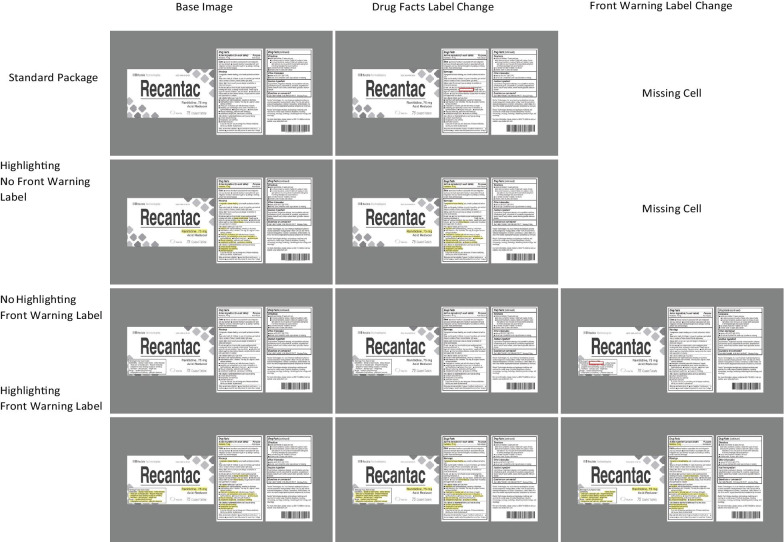
Examples of the six warning changes for a given brand. The left column depicts the complete label for the 4 label treatments. The middle depicts the altered images with a change to the Drug Facts Label. The right depicts the altered images with a change to the front warning label. The red squares in the top row are to illustrate the change location and did not appear in the experiment

For warning changes, the complete factorial design used for active ingredients was impossible; a change in warnings on the PDP can occur only in conditions with our novel front warning label; warning information does not occur on the front of the package in standard labels. As a result, there were two missing cells in the overall 2 (location of change: PDP vs Drug Facts Label) × 2 (front warning label vs standard label) × 2 (highlighting vs no highlighting) design (see Fig. 4). Given this reduction in warning conditions, and our focus on evaluating our novel designs using a front warning label and highlighting, we doubled the number of warning label changes. For each mock brand we identified two warnings to change. For each of these two warnings, the six conditions of the design were created, resulting in a total of 36 warning changes across the three mock brands.

The warning changes involved the appearance and disappearance of one of the warnings phrases that was selected for inclusion on the front warning label (e.g., “stomach ulcers or bleeding”). It is important to note that the exact same text appeared and disappeared for the six trials with a given warning; the font size, phrase size, and word complexity were held constant. In highlighted conditions both the highlighting and text appeared and disappeared.

Experiment 2 was similar to Experiment 1 with the following differences. First, we developed a new set of four mock brands, in order to increase the generalization across stimuli. Second, we were somewhat concerned that the over sampling of warning information in Experiment 1 may have created a bias toward warning information based on history effects (Awh et al., 2012). So, for each mock brand we had a single warning that changed. Third, we significantly shortened the experiment, and counterbalanced the full design across sets of two participants. This was done because participants in Experiment 1 complained about the length of the task, and Experiment 2 targeted a more at high-risk, lower educated population who we postulated might have increased difficulty with the task and, as such, experience fatigue and withdrawal from the experiment.

As a result, in experiment 2, each participant only performed a total of 64 trials, comprised of 28 critical and 36 non-critical trials. The non-critical trials were designed to achieve the same three goals of the non-critical trials discussed for Experiment 1. The critical trials consisted of 16 changes to active ingredient information, and 12 warning changes. Across two participants there were a total of 32 active ingredient changes; the full factorial of the 4 (mock brands) × 2 (location of change PDP or Drug Facts Label) × 2 (label type: front warning label or standard) × 2 (highlighting/no highlighting) design.^2^ Across the same two participants there were a total of 24 warning changes; the full factorial design less the missing cells caused by the fact that changes could not occur on the package’s front when there was a standard label.

An additional difference of note between the two experiments was that the front warning label was located in the lower left of the PDP in Experiment 1 but was presented on the lower right for Experiment 2. The front warning label was moved to the lower right in experiment 2 to better match the design of commercially available labels, in which Active Ingredient and indications information is generally left justified on the PDP. Finally, in Experiment 2 when there was a change to highlighted information, only the highlighting disappeared (the text remained), in order to better examine the effect of the presence of highlighting.

### Procedure

Prior to participation in the experimental task, participants were characterized in a number of ways. The first was the Short Blessed Test (Katzman et al., 1983), a screener for cognitive impairment consistent with dementia. Scores of 9 or more are indicative of cognitive impairment; as such, participants who scored a 9 or above were deemed unable to provide informed consent, and were thanked for their time, provided the $50.00 incentive and dismissed from the study without further data collection. Those continuing completed a survey that included: basic demographics (age, gender, race and ethnicity, native language, annual income, and educational attainment). Participants also performed a health literacy screening [REALM-R (Bass et al., 2003)], near point visual acuity test (Sloan Pocket Size Near Vision Card with Continuous Text by Precision Vision in Woodstock IL) and color vision assessment (Pseudo-Isochromatic Plates by Richmond Products, Southeast Albuquerque NM).

After completing the section of the testing intended to characterize them, participants began the main task. They were instructed to look for changes between the flickering displays and signal that they detected the change by pressing the space bar as soon as they located it. When the participants pressed the space bar, the reaction time timer was stopped, and the images stopped flickering (on the original image). At that point participants made an un-timed, mouse click on the location of the change, in order to verify that they had correctly identified the change. After receiving instructions, participants performed four practice trials to acquaint them with the task and provide time for questions/clarification related to the experiment. It also provided the research team with the opportunity to ask participants who did not seem comfortable with the mouse if they would like to just point at the screen to indicate the location where they saw the change and have a researcher mouse over this location for them. If the participant clicked on the change (± ~ 1°) the trial was coded as a “hit”. Trials where the change was not correctly located or was not found prior to trial time out at 18 s were coded as “misses.”^3^ Each mouse click (indicating the change location) began the next trial. This process continued until all trials were completed.

### Data analysis

We analyzed the changes in the active ingredients separately from the changes in warnings. One reason for this decision was that the design was unbalanced, with two missing cells for the standard treatments; due to the current regulations and practice (discussed previously), conditions without our novel front warning labels did not have changes to a warning in the PDP, because warning information never appears there. In addition, we anticipated that the addition of the front warning label should increase attention to warning information, but might actually draw attention away from the active ingredient information (which appeared in its typical spot—on the PDP—not in the boxed warning we added—see Fig. 1). Finally, based on regulations and current commercial practice, the active ingredient on the PDP typically appears in much larger font than the warnings, thus directly comparing across those types of changes would be confounded by differences in the size of the changes.

Our two dependent variables were reaction times (RT) for correct change detections (hits) and accuracy. Given the positive skew of the RT measure, the RT data were log transformed prior to analyses. We report all RT statistical results in these log transformed units. However, for illustrative purposes the figures present means and standard errors of the raw RT data in seconds. In addition, means and standard deviations in seconds are presented in Table 1.

**Table 1:** Reaction times and standard deviations in seconds for all conditions in Experiments 1 and 2

		Change to PDP		Change to drug facts label	
		Highlighting	No highlighting	Highlighting	No highlighting
*Active ingredient changes*					
Exp 1	Front warning label	4.12 (2.06)	5.80 (3.44)	6.83 (3.11)	7.76 (3.43)
	Standard label	3.86 (2.69)	4.88 (3.35)	6.38 (2.52)	7.27 (3.68)
Exp 2	Front warning label	5.08 (2.49)	6.27 (3.62)	8.18 (3.31)	8.58 (3.07)
	Standard label	4.28 (2.76)	5.37 (2.71)	7.06 (3.03)	7.95 (3.82)
*Warning changes*					
Exp 1	Front warning label	5.74 (2.85)	6.63 (3.23)	9.85 (2.7)	11.28 (3.)
	Standard label			9.63 (2.77)	11.24 (3.39)
Exp 2	Front warning label	8.21 (4.67)	8.84 (4.15)	11.28 (4.36)	12.12 (3.15)
	Standard label			11.37 (2.82)	12.57 (3.38)

Our initial data analytic approach was to perform the analyses using multilevel models that included both fixed and random effects for each of the independent variables. However, our analyses indicated that there was insufficient random variation to estimate models with random effects for the independent variables.^4^ Thus, active ingredient RT data were analyzed using multilevel modeling (MLM) treating location of change (the PDP or the Drug Facts Label), highlighting (present or absent), and label type (standard or our novel front warning label) as categorical predictors in a 2 × 2 × 2 factorial design that included all main effects and interactions as fixed effect predictors. We also included participant age and RT from the non-critical trials as covariates.^5^ The repeated measures nature of the data was modeled using compound symmetry, which (like standard within-subjects ANOVA designs) imposes an assumption of homoscedasticity. This approach is equivalent to including a random intercept for the subject effect, making the analysis functionally equivalent to a within subjects ANOVA with covariates. However, performing the analysis as an MLM is more appropriate given that we had unbalanced data (i.e., we had unequal numbers of observations across subjects because RT was analyzed only for correct trials).

The warning RT data were also analyzed using MLM with the same covariates (RT from non-critical trials and participant age) and repeated measures model. However, given the unbalanced design for changes to warning information, the analysis was a 3 (conditions: front warning label with a change to the PDP, front warning label with a change to the Drug Facts Label, and Standard Labels with a change to the Drug Facts Label) × 2 (highlighting/no highlighting) design.

Analyses of the accuracy data were conducted in a parallel fashion with the exception that the dichotomous nature of the outcome required that we use binary logistic MLM. As with the RT data, separate analyses were used for the active ingredient changes (the 2 × 2 ×2 model) and warning changes (the 3 × 2 model). Models also included age as well as the number of correct responses on non-critical trials as covariates. As with the RT data, preliminary analyses indicated that there was insufficient random variation in the outcome to be able to estimate a complex random effects model that included the predictors. Therefore, the same repeated measures approach as was used for RT was used for accuracy.

## Results

After screening for inability to provide informed consent and removing incomplete data, 60 participants were included in the analysis of Experiment 1 and 57 participants in Experiment 2, respectively. In experiment 2, 1 person was dropped due to their Short Blessed Test Score being higher than 8, and 2 people were dropped due to computer errors leading to the subjects withdrawing before completion of the study task. The sample for Experiment 1 included 37 women and 23 men who identified as White not-Hispanic (*N* = 52), African-American not Hispanic (*N* = 6), and Hispanic (*N* = 2). The mean age was 70.37, SD = 4.59 and education levels varied with 12 individuals with High-school degrees, 32 with some college up to a bachelor’s degree, and 16 with post-graduate education. The final sample for Experiment 2 included 40 women and 17 men with 46 participants who were White non-Hispanic and 8 who were African-American not Hispanic. Three individuals did not provide this information. The mean age was 71.04, SD = 6.95 and education levels varied with 23 individuals with High-school degrees or less, 25 with some college up to a bachelors degree, and 9 with post-graduate education. A chi-square test indicated that participants in Experiment 2 had significantly less education than those in Experiment 1 (*p* = 0.045).

### Active ingredient changes

#### Experiment 1

**Fig. 5:**
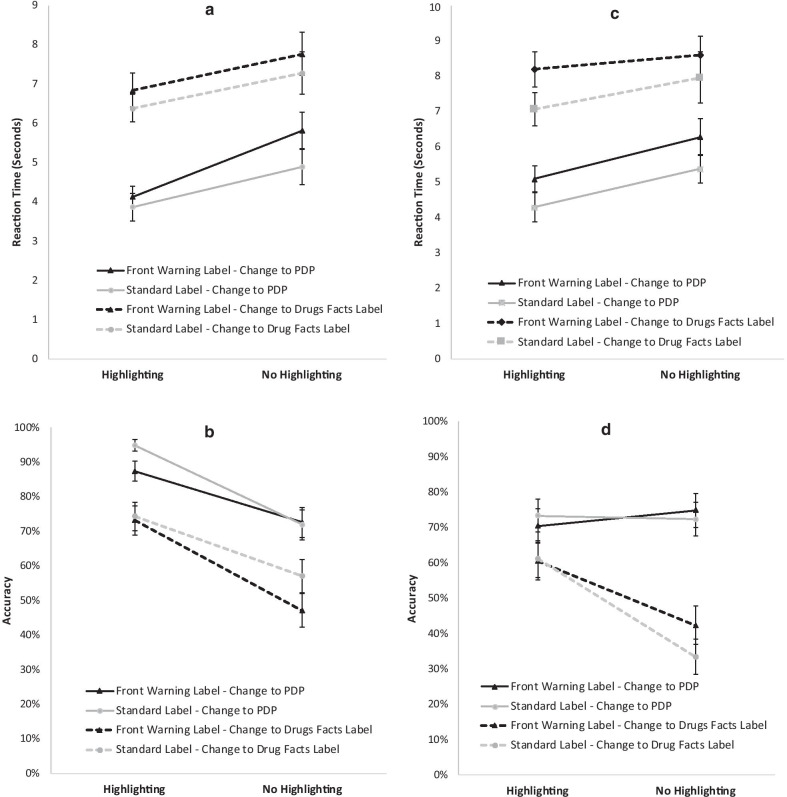
**a** Presents the mean reaction time for changes to active ingredients by condition for Experiment 1. **b** The corresponding mean accuracy data. **c**, **d** Present the mean reaction time and accuracy data for changes to active ingredients by condition for Experiment 2. For accuracy the values are the estimated marginal means. For reaction time, rather than present the log transformed data that was used for the analyses, for illustrative purposes we present the means of the raw data. Error bars represent the standard error of the mean

The analysis of rection time data (see Fig. 5a) found a main effect of label treatment, *F*(1, 419) = 7.4, *p* = 0.007, *d* = 0.10. Consistent with our hypothesis that a front warning label would attract attention to itself that could potentially compete with the active ingredient information for attention, changes to active ingredients were found more slowly in treatments containing a front warning label (*M* = 3.72, SE = 0.03) than those without the additional warning label (*M* = 3.66, SE = 0.029). There was also a main effect of Highlighting, *F*(1, 422) = 16.17, *p* < 0.001, *d* = 0.15, with faster change detections for treatments that included highlighting (*M* = 3.64, SE = 0.029) than those without (*M* = 3.74, SE = 0.03). The main effect of location of change (PDP vs Drug Facts Label—see Fig. 1), *F*(1, 435) = 75.3, *p* < 0.001, *d* = 0.33, was also significant, with faster change detection when changes in critical information occurred on the PDP (*M* = 3.58, SE = 0.029) than on the Drug Facts Label (*M* = 3.8, SE = 0.03). None of the two or three-way interactions approached significance, all *p* > 0.21.

These patterns were echoed in the accuracy data (see Fig. 5 B). There was a main effect of label, *F*(1, 1430) = 5.75, *p* = 0.017, *d* = 0.09. Consistent with the previous analysis of RT results, accuracy data suggested that a front warning label diverted attention from the active ingredient information; accuracy was lower for treatments with a front warning label (*M* = 72%, SE = 2.9%) than for treatments that did not contain one (*M* = 78.5%, SE = 2.7%). The main effect of highlighting was also significant, *F*(1,1430) = 63.20, *p* < 0.001, *d* = 0.31, with more accurate change detection rates for treatments with highlighting (*M* = 84.9%, SE = 2.2%) than without it (*M* = 62.7%, SE = 2.1%). Location also rose to significance, *F*(1, 1430) = 55.74, *p* < 0.001, *d* = 0.29, with higher accuracy for changes in critical information on the PDP (*M* = 84.2%, SE = 2.3%) than the Drug Facts Label (*M* = 63.6%, SE = 3.1%). Here, the three-way interaction also reached significance, *F*(1,1430) = 5.24, *p* = 0.022. Breaking down this interaction by label treatment so that we can examine the change location (PDP vs Drug Facts Label) by highlighting (present and absent) interactions separately by label treatment (standard and front warning label), the change location by highlight interaction is significant for standard labels, *F*(1,714) = 7.097, *p* = 0.008, but not for those including the novel front warning labels, *F*(1,714) = 0.177, *p* = 0.674. Examination of the means and simple effects tests indicate that although responses are more accurate with highlighting than without, the advantage of highlighting is greater for changes in the PDP location *F*(1,356) = 37.34, *p* < 0.001, *d* = 0.44, than for changes in the Drug Facts Label, *F*(1,356) = 11.13, *p* = 0.001, *d* = 0.30. The advantage in terms of increased accuracy when stimuli are highlighted for changes to the PDP was 0.247 and the advantage of highlighting for changes to the Drug Facts Label was 0.189. This is not terribly surprising since the font of the active ingredient, and thus the amount of highlighting, is larger in the PDP location than when changes in this information occurred in the Drug Facts Label.

#### Experiment 2

The pattern of data in Experiment 2 was similar to that of the original experiment (see Fig. 5c, d). Like Experiment 1, all three main effects were significant. As with the first experiment, the main effect of label treatment, *F*(1, 489) = 10.03, *p* = 0.002, *d* = 0.18, was significant with the front warning label competing with the active ingredient for attention, resulting in faster detection of active ingredient changes in treatments without a novel front warning label (*M* = 3.70, se = 0.021) than those with the front warning label (*M* = 3.77, SE = 0.021). There was also a main effect of highlighting, *F*(1, 506) = 9.82, *p* = 0.002, *d* = 0.18, with faster change detections in highlighted treatments (*M* = 3.7, SE = 0.021) than those without (*M* = 3.77, SE = 0.022). There was also a significant effect of location, *F*(1, 511) = 135.8, *p* < 0.001, *d* = 0.68, with faster change detection when the change occurred in the PDP (*M* = 3.61, SE = 0.02) than in the Drug Facts Label (*M* = 3.85, SE = 0.022). However, in contrast to Experiment 1, a significant highlighting by location interaction, *F*(1, 496) = 4.42, *p* = 0.036 was indicated for Experiment 2. The source of the interaction was that highlighting had a stronger effect on response times (Highlighted treatments were detected more quickly) for critical information changing in the PDP than for critical changes that occurred in the Drug Facts Label. For trials where the change occurred to critical information on the Drug Facts Label, the simple effect of highlighting was not significant, *F*(1,204) = 0.45, *p* = 0.503, but when the change occurred on the PDP, performance was faster on trials with highlighted labels, *F*(1,286) = 14.43, *p* < 0.001, *d* = 0.37.

For the accuracy data, the main effects for highlighting, *F*(1, 894) = 8.57, *p* = 0.004, *d* = 0.14, and location, *F*(1,894) = 47.05, *p* < 0.001, *d* = 0.33, were statistically significant, echoing the reaction time data. Changes in highlighted treatments were more likely to be detected (*M* = 66.5%, SE = 3.3%) than treatments that were not highlighted (*M* = 56.5%, SE = 3.7%). Accuracy was also higher for changes in the PDP (*M* = 73.7%, SE = 3.1%) than changes in the Drug Facts Label (*M* = 49.2%, SE = 3.6%). However, the main effect of label treatment did not approach significance, *F*(1, 894) = 0.344, *p* = 0.56. So, unlike the reaction time data, the presence of our novel front warning label did not impact the ability to accurately detect changes to the active ingredient information. Like with reaction time, there was a significant highlighting by location interaction, *F*(1, 894) = 12.32, *p* < 0.001, however the source of this interaction differs from the source for reaction time. For changes to the PDP accuracy was high for both highlighted and nonhighlighted conditions; there was no effect of highlighting, *F*(1,443) = 0.158, *p* = 0 0.691. In contrast, for changes in the Drug Facts Label, participants were more accurate with highlighted than nonhighlighted treatments, *F*(1,449) = 24.28, *p* < 0.001, *d* = 0.44.

## Discussion of active ingredient changes

The data across both experiments was highly consistent, supporting three general conclusions. First, *highlighting effectively draws attention to itself;* changes to critical information were detected more quickly and/or more accurately in highlighted treatments. Second, we have clear evidence that *the addition of a front warning label enhances attention to critical warnings*; that said, the addition of such a label competes with other critical information, namely the active ingredient, for attention. Changes to the active ingredient information were detected more slowly and/or less accurately in the presence of the competing, novel warning label (recall that the active ingredient did not appear in the warning label). Finally, it is clear that *observers were faster and more accurate at detecting active ingredient changes that occur on the PDP than on the Drug Facts Label*. One possible interpretation of this result is that observers have a bias to attend to information on the PDP, an interpretation that is consistent with work on nutrition labels (Bix et al., 2015). However, because we followed regulations and commercial practice to create realistic treatments, we continued to format the active ingredient in a typical placement and font size on the PDP which was not size (or font) matched to the active ingredient information in the Drug Facts Label, limiting the strength of this conclusion.

### Warning label changes

#### Experiment 1

**Fig. 6:**
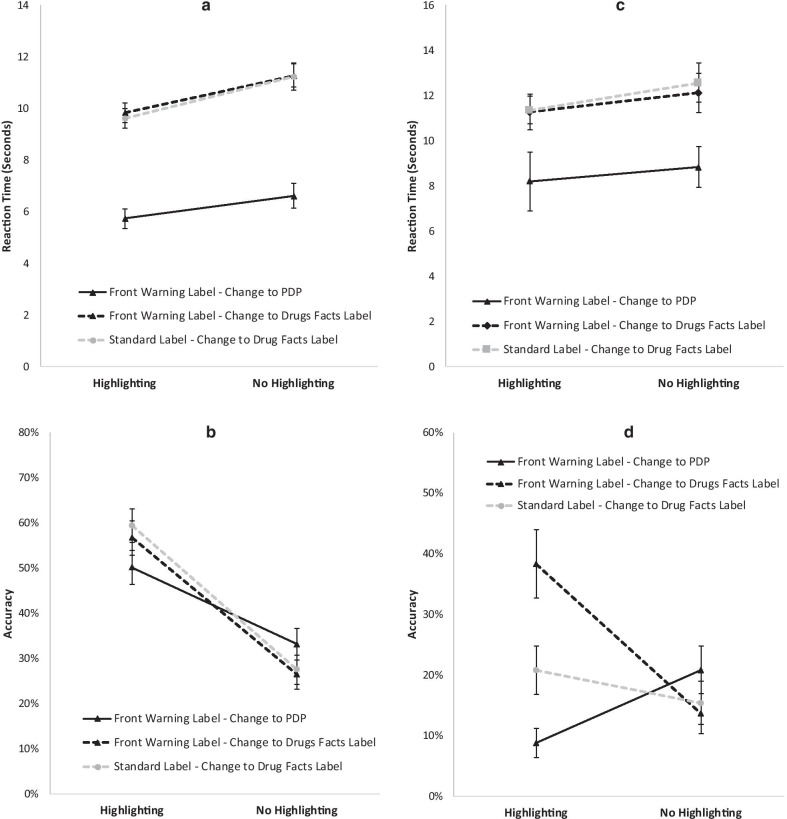
**a** The mean reaction time for changes to warnings by condition for Experiment 1. **b** The corresponding mean accuracy data. **c**, **d** present the mean reaction time and accuracy data for changes to warnings by condition for Experiment 2. For accuracy the values are the estimated marginal means. For reaction time, rather than present the log transformed data that was used for the analyses, for illustrative purposes we present the means of the raw data. Error bars represent the standard error of the mean

The mean reaction times by condition are plotted in Fig. 6a. Given that there was not a full factorial design (no changes to warnings positioned on the PDP were possible in the standard label conditions), we ran an omnibus 3 (Conditions: novel front warning label with a change to PDP, front warning label with a change to the Drug Facts Label, and standard label with a change to the Drug Facts Label) × 2 (highlighting/no highlighting) analysis. There was a significant main effect of condition, *F*(2,448) = 142.733, *p* < 0.001, a main effect highlighting, *F*(1,453) = 11.676, *p* = 0.001, and no significant interaction, *F*(2,445) = 1.225, *p* = 0.295. The main effect of highlighting indicates that changes were detected more quickly for highlighted (*M* = 3.823; SE = 0.024) than unhighlighted (*M* = 3.896; SE = 0.027) conditions, *d* = 0.13. To localize the source of the main effect of condition we performed Bonferroni adjusted pairwise comparisons. These demonstrate that the condition which had our novel front warning label and a change to the PDP (*M* = 3.608, SE = 0.027) differed significantly from both the condition with a novel front warning label with a change to the Drug Facts Label (*M* = 4.001, SE = 0.027, *d* = 0.82) and the standard label with a change to the Drug Facts Label conditions (*M* = 3.970, SE = 0.028, *d* = 0.75). The latter two conditions did not differ, *d* = 0.06.

The same 3 × 2 analysis on accuracy (see Fig. 6b) found no main effect of condition, *F*(2,2152) = 0.328, *p* = 0.721, a significant effect of highlighting, *F*(1,2152) = 151.687, *p* < 0.001, and a significant interaction, *F*(2,2152) = 5.510, *p* = 0.004. The interaction occurred because, although the effect of highlighting was significant for each condition, it was less pronounced for the condition with a the novel front warning label and a change to the PDP (*F*(1,2152) = 22.403, *d* = 0.25) than the other two conditions (novel front warning label with a change to the Drug Facts Label *F*(1,2152) = 68.798, *p* < 0.001, *d* = 0.45; Standard Label with a change to the Drug Facts Label *F*(1,2152) = 74.991, *p* < 0.001, *d* = 0.47). The lack of a main effect of condition, suggests that the RT results are not a result of a speed/accuracy tradeoff. The main effect of highlighting was consistent with reaction time findings, with higher accuracy for the highlighted (*M* = 0.554; SE = 0.031) than non-highlighted conditions (*M* = 0.289; SE = 0.028, *d* = 0.27).

Experiment 2. The mean reaction times by condition are plotted in Fig. 6c. We performed the same 3 × 2 analysis as for Experiment 1 on the RT data. There was a main effect of condition, *F*(2,121) = 11.628, *p* < 0.001, but the highlighting main effect did not reach significance, *F*(1,126) = 2.145, *p* = 0.145, nor did the interaction, *F*(2,120) = 0.206, *p* = 0.814. Bonferroni adjusted pairwise comparisons to localize the main effect of condition, again, found that the condition with the front warning label, and a change in the PDP location (*M* = 3.870, SE = 0.031) was detected more quickly than either of the other two conditions, (front warning label with change to the-Drug Facts Label *M* = 4.042, SE = 0.028, *d* = 0.87; Standard Label with a change to the Drug Facts Label *M* = 4.051, SE = 0.029, *d* = 0.92). Those later two conditions did not differ significantly, *d* = 0.05.

The same 3 × 2 analysis on accuracy data (see Fig. 6d), found a main effect of condition *F*(2,677) = 3.895, *p* = 0.021, as well as a significant interaction between condition and highlighting, *F*(2,677) = 11.904, *p* < 0.001. The effect of highlighting was not significant, *F*(1,677) = 1.625, *p* = 0.203. Bonferroni corrected pairwise tests indicate that the condition with a front warning label and change in the Drug Facts Label (*M* = 0.238, SE = 0.036) had higher accuracy than the condition with a front warning label and a change to the PDP (*M* = 0.137, SE = 0.025, *d* = 0.22). Neither of these two conditions were significantly different from the standard label (*M* = 0.179, SE = 0.029, both ds < 0.14). The interaction occurred because there were substantial differences in accuracy as a function of condition when changes were highlighted, *F*(2,677) = 13.864, *p* < 0.001, (standard vs. drugs facts *d* = 0.43, standard vs. PDP *d* = 0.30, drug facts label vs PDP *d* = 0.73) but not in the absence of highlighting, *F*(2,677) = 1.330, *p* = 0.265, all *d*s < 0.18.

### Discussion of warning changes

The Reaction time Data from Experiment 1 provide strong evidence for a bias to attend to information on the PDP; reaction times were much faster when a change to the critical warning information appeared on the PDP than when the warning information changed in the Drug Facts Label. This reaction time advantage for changes to the PDP was replicated in the second experiment. It is important to note that in these comparisons, unlike in the active ingredient changes, the warnings that changed were identical in both locations and, thus, not attributable to differences in size or change complexity. Further, in Experiment 1 this difference cannot be attributed to a speed accuracy trade off. However, in Experiment 2, the accuracy for the changes to front of pack warnings was surprisingly low, raising the possibility that the replication of the reaction time benefit for front of pack labels could have been a result of a speed accuracy tradeoff.

Experiment 1 also showed a fairly clear benefit of highlighting, which is consistent with the results from the active ingredient changes. However, it is worth noting that the effect of highlighting was more prevalent on accuracy than reaction time in Experiment 1. In Experiment 2, where accuracy was generally low, the effect of highlighting did not replicate. In short, with the exception of the accuracy data for experiment 2, all the data from the warning labels and the active ingredient changes, suggest that both highlighting and front of pack warnings are effective at garnering attention to critical information. The reasons for the anomalous accuracy data in Experiment 2 will be further discussed in the general discussion.

## General discussion

We set out to address two specific aims focused on how novel label designs for OTCs impact attentional allocation to critical information by older adults. *Overall, these two experiments suggest that both highlighting and adding a front of package warning label have the potential to increase attentional allocation to critical OTC medication information viewed by older adults*. Both experiments provide evidence that use of a label that highlighted critical information resulted in more attention to that critical information than an unhighlighted label. Highlighting led to an increase in accuracy and a decrease in RT for active ingredient information in both experiments. Highlighting also resulted in increased accuracy and decreased reaction time for changes to critical warnings in Experiment 1. The only case where highlighting did not show consistent attentional benefits was in the warning changes of Experiment 2, an anomalous case that will be discussed below. In addition, *our results provide evidence that information on the PDP was more likely to be attended to than information in the Drug Facts Label*. This finding suggests that the addition of a front warning label is a strategy worth pursuing as a means to increase awareness of critical warnings. However, we note that there is a tradeoff; the increased attention to warnings produces a slight reduction in attention to the active ingredients in the PDP location. However, we note that performance for the currently used label (standard with no highlighting see Fig. 2) was far superior for active ingredients than warnings, so we believe the benefits of increasing attention to the warnings will outweigh the costs of diverting some attention away from active ingredients.

Further, we believe that the advantages our study enumerates regarding the use of a novel front warning label are actually underestimated by our study design. Specifically, in order to employ the change detection methodology, stimulus material was presented as flattened designs which included both the Drug Facts Label and the PDP simultaneously (see Fig. 1). The advantage of the front warning label is likely to be even more pronounced in realistic contexts where the box would have to be rotated to view the information present on the Drug Facts Label; eye-tracking research investigating attentional behavior when evaluating an OTC package suggest that people often do not even turn to the side of the package containing the Drug Facts Label (Liu, 2016).

In general, both experiments suggest that both of our label treatments are promising strategies and the patterns of data from both experiments were highly consistent. However, there was one exception; the accuracy data for highlighted warning changes in Experiment 2 were surprisingly low and did not follow this pattern. While these findings might raise questions about the strength of our conclusions, we think the highly consistent findings across all conditions suggest that instead there may be something anomalous occurring in these conditions. While it is speculative, we think the low accuracy rates in these conditions occurred because of how we implemented the change in these conditions. In these conditions, the change involved only highlighting disappearing from the warnings, the warning text did not change. By contrast, in Experiment 1, both the highlighting and warning text disappeared. Thus, one plausible interpretation of the very low accuracies in the highlighted conditions of Experiment 2 is that highlighting serves to draw attention to the highlighted text, without people necessarily encoding the highlighting itself. Thus, highlighting could make people more sensitive to changes in highlighted content, as they were in Experiment 1, even though people were relatively insensitive to detecting that the highlighting itself was changing across flicker. In some respects, this explanation is similar to the finding of one of the earliest reports of change blindness. McConkie and Zola (1979) had participants read text that was presented in AlTeRnAtInG tExt (McConkie & Zola, 1979). During some eye movements, the case of every letter in the passage changed, yet none of the subjects noticed this massive change to the physical properties of the text. Instead readers seemed to be encoding the words to some deeper level (e.g., letter identification or semantics) which are not influenced by the change in the physical properties.

It is also worth noting that overall accuracy, even in the other conditions was lower in Experiment 2 than in Experiment 1. While there are many reasons this could have occurred, one possible reason is that the demographics of the subjects differed between the experiments. Participants in Experiment 1 were more highly educated than participants in Experiment 2. These factors may have made the task overall more difficult for the participants in second experiment.

## Limitations

To assess our ability to detect potential differences between our experimental treatments, we conducted a series of post-hoc sensitivity power analyses using G*Power (Faul et al., 2007) for a factorial repeated measures design with either 6 (warning changes) or 8 (active ingredient changes) cells in which the correlations for repeated measures ranged from 0.10 to 0.20 (which were the observed values from our data), and the sample size was 60 (Exp 1) or 57 (Exp 2). These analyses indicated that using an alpha of 0.05 and power of 0.80 our samples were powered to detect effects ranging in size from *d* = 0.28 to 0.38. Thus, our analyses could detect moderate effects. It is possible that some of the effects that did not emerge as statistically significant were smaller and may have been found if the sample sizes for the two experiments had been larger. Even so, across experiments our results provide compelling evidence that attention was prioritized to information on the front of the package and to highlighted information.

A second possible limitation is that for changes involving the active ingredient, the comparison between changes to the front of pack and the Drug Facts Label had a confound because the active ingredient appeared in larger text when on the front of the package. While this confound is undesirable from a methodological perspective, it is necessary when performing this type of real-world research. In practice the active ingredient on commercially available products is presented at a much large front on the front of the package than in the Drug Facts Label. We intentionally mimicked this reality in our design. Further, we note that this size confound did not occur in the warning changes, and there was a clear RT advantage for front of pack changes in these conditions as well. Thus, we do not believe that the size confound can completely explain our results. Instead, consistent with prior research (Becker et al., 2015; Graham et al., 2015; Liu, 2016), we believe these results suggest that information appearing on the front of packages is prioritized for attention.

An additional possible criticism is that prior research has already established that highlighting and repositioning warnings to the front of the package would induce attention. We tested these approaches because they held the promise of benefit, however there was no guarantee that they would be effective. Literature exploring the effects of highlighting text is somewhat mixed. While much of this research has been done in the context of examining effective study habits, it suggests that highlighting may be ineffective if too much information is highlighted (Dunlosky et al., 2013). Given the number of warning that are deemed critical to reducing ADRs across individuals, there was a real concern that the amount of highlighting, and such a dense area of highlighting, in the front warning label might counteract its possible benefits. Thus, determining the efficacy of highlighting all the drug/drug and drug/diagnosis warnings that exist for currently available OTCs is of value. Further, while prior work in nutritional labeling supports the use of front-of-pack labelling ( see Kanter et al., 2018 for a review), there is limited evidence of its effectiveness for OTC medications (Liu, 2016). In addition, we note that there is a good deal of inertia against the incorporation of these ideas into regulations (front of pack nutrition labels are yet to be standardized or required for US markets), and, absent regulation, manufacturers are reticent to adopt. Growing the available body of objective evidence is an important step in informing and catalyzing regulations to overcome this inertia. The data we provide are a first piece of evidence to suggest that these types of label changes may be beneficial, however, we recognize that it is just one piece of the evidence that will be needed to influence change.

A related substantive concern is whether results from a change detection method would generalize to a purchase scenario. While this is a legitimate concern, there are both reasons why we believe it may generalize and reasons why it is important to do these type of controlled studies before trying to implement a more realistic intervention. Prior work that compared verbal descriptions of scenes to change detection performance (Rensink et al., 1997), suggested that the same elements that were prioritized during the verbal descriptions, where those that were detected quickly during change detection. That is, across very different tasks (a change detection and verbal description task) the elements that were prioritized for attention remained consistent. In addition, as mentioned in the introduction, research on nutrition labeling shows that labels indicated to be prioritize for attention via a change detection method (Becker et al., 2015) were also prioritized during product evaluation and selection as indicated by eye tracking (Bix et al., 2015; Graham et al., 2015; Van Herpen & Van Trijp, 2011). This gives us some confidence that the attentional prioritization metrics determined by our change detection task would generalize across disparate tasks.

That said, we note that the gold standard for evaluating whether these label changes will be effective is to engage in a naturalistic experiment in which they are implemented within a store context, and changes in purchasing behavior associated with their introduction are investigated. However, we note that this type of gold standard rarely occurs, because it is prohibitively expensive and difficult to implement. In addition, prior to embarking on such a trial, it is important to first verify that the label one implements during such a trial is a label that is likely to be effective; testing designs that garner attention are critical given that attending to critical information is a prerequisite to further processing of that information. In the absence of attention, the critical messages communicated by the label will be derailed at an early stage of processing.

Indeed, warnings on cigarette packages provide a prime example of why designing a label that is likely to attract attention before widespread implementation is critical. There have been different methods of labelling the dangers of smoking on cigarette packages throughout the world, with the US adopting a small, black and white, text-based warning that appears on the side of the package and Canada adopting a large picture and text-based warning on the front of the package. A 2006 study (Hammond et al., 2006) of the effectiveness of these warnings, found that frequent smokers (> 16 cigarettes per day on average) in Canada were almost twice as likely as their US counterparts to report noticing information about the dangers of smoking from cigarette packages, with over 53% of US smokers failing to report noticing the warning often or very often in the last 6 months. Importantly, the study also found that noticing warning on cigarette packages was positively associated with knowledge of the health risks of smoking, even after adjusting for noticing anti-smoking messages in other media. That is, there is evidence from the labeling of cigarettes that labels can be an effective source of information, but their ability to provide this information depends critically on designing a label that will garner attention.

We note that the type of pictorial warning used for cigarettes is impossible to implement with OTCs given that OTC warnings are more extensive and which warning is most important often depends on the particular observer’s health status. Even so, the example highlights the importance of doing pretesting of a label to establish that is likely to attract attention to itself, prior to under-going such a trial implementation.

## Future directions

As with any single study, the conclusion we draw require replication and ideally those replications will provide converging measures and disparate tasks to help further the generalizability of these results. Additionally, going forward it will be important to determine whether these same types of labels are also particularly beneficial when people are trying to evaluate the safety of medications, and engaging their volitional attentional systems. To do so, we have begun collecting data (which was suspended mid-collection due to Covid-19) on experiments that use more traditional visual search tasks in which older adults are given the goal of searching these labels for a specific warning or active ingredient. We also have planned experiments in which participants are asked to evaluate whether a given OTC medication would be appropriate for them to take given their health status, which should allow us to evaluate how these different labels influence decision making. Finally, we aim to integrate eye-tracking as well as reaction time and accuracy measures. We believe that across these tasks we will be able to comprehensively evaluate how these types of label designs impact attention and decision making, in hopes of providing well-supported recommendations for improved OTC labels which mitigate the likelihood of an ADR. As noted above, these types of lab-based, controlled studies are an important precursor to larger scale naturalistic interventions in real-world contexts; prior to launching such an intervention it is necessary to ensure that the label one is introducing is likely to be successful at garnering attention to itself.

## Conclusion

In summary, the results of the two change detection studies suggest that highlighting information critical to the safe and effective use of OTC drugs and the use of a front of package warning label are beneficial in attracting older adults’ attention. These two labeling strategies should be further explored and if our findings are found to be robust across tasks and converging measures, regulators interested in increasing the likelihood consumers interact with critical safety information before making OTC purchase should consider these factors for implementation.

In closing, while we have focused on OTC labeling in this paper, we believe that there are myriad of opportunities for people who study visual cognition to apply their knowledge and methods to the design and evaluation of more effective methods of conveying critical information to consumers and health care providers. By leveraging those techniques and knowledge, it may be possible to substantially increase health and safety.

## Data Availability

All stimuli and datasets used and/or analyzed for the current study are publicly available on the Open Science Framework at https://osf.io/s8h54/.

## Footnotes

1 The same regulation mandated that “NSAID” be highlighted or bolded for the group of active ingredients classified as Non-steroidal Anti-inflammatory Analgesic, Antipyretic Drugs. However, the practice of highlighting the category NSAID has not yet been widely implemented on commercial packaging, rather manufacturers are opting for the option to bold the text.

2 During analyses we realized that for one of the two versions of the experiment designed to completely counterbalance factors across a pair of subjects, one trial that was supposed to contain a change to an active ingredient mistakenly made a change to a warning. So for half the subjects, there was one additional warning change and one fewer active ingredient change in the condition with a front of pack warning that was non-highlighted where the change occurred on the PDP.

3 In both experiments a vast majority of “misses” were time-out (> 80% in both experiments) rather than mis-locations of the change.

4 ^4^Our initial data analytic models included fixed effects for all main effects and interactions between location change, highlighting, and label type, and it included random slopes for all main effects and interactions along with the specification of compound symmetry to model the residual variances. The random slopes allowed person to person variation in each of the main effects and interactions. However, this complex random effects model did not result in an admissible solution for either accuracy or RT. We therefore first dropped all of the interactions from the random effects and specified the full fixed effect model along with a random slopes model that included only the main effects for location change, highlighting, and label type, and the repeated compound symmetry specification for the residuals. This simplified model also produced non positive definite Hessian matrices for RT and accuracy. We again simplified the random effects to check whether a model specifying a random slope for only one of the three main effects was estimable. None of the three models was estimable for either RT or accuracy. Thus we used the model that included the 2 × 2 × 2 specification for the fixed effects and no random slopes for the predictors. The implication of the fact that the random slopes models could not be estimated is that the effects of the independent variables (main effects and interactions) were relatively consistent from person to person and so the effects of the independent variables were well summarized by the fixed effect (i.e., mean) estimates and there was little evidence of systematic individual difference variation in the modelled effects.

5 We conducted these (and all other) analyses dropping the noncritical trial score as a covariate and results did not change.
